# Genomic analysis of halophilic bacterium, *Lentibacillus* sp. CBA3610, derived from human feces

**DOI:** 10.1186/s13099-021-00436-2

**Published:** 2021-06-23

**Authors:** Seung Woo Ahn, Se Hee Lee, Hong-Seok Son, Seong Woon Roh, Yoon-E Choi

**Affiliations:** 1 Microbiology and Functionality Research Group, World Institute of Kimchi, Gwangju, 61755 Republic of Korea; 2 Department of Food Bioscience and Technology, Korea University, Seoul, 02841 Republic of Korea; 3 Division of Environmental Science and Ecological Engineering, Korea University, Seoul, 02841 Republic of Korea

**Keywords:** *Lentibacillus* sp. CBA3610, Complete genome sequence, Gut microbiota, Halophile

## Abstract

**Background:**

*Lentibacillus* species are gram variable aerobic bacteria that live primarily in halophilic environments*.* Previous reports have shown that bacteria belonging to this species are primarily isolated from salty environments or food. We isolated a bacterial strain CBA3610, identified as a novel species of the genus *Lentibacillus*, from a human fecal sample. In this report, the whole genome sequence of *Lentibacillus* sp. CBA3610 is presented, and genomic analyses are performed.

**Results:**

Complete genome sequence of strain CBA3610 was obtained through PacBio RSII and Illumina HiSeq platforms. The size of genome is 4,035,571 bp and genes estimated to be 4714 coding DNA sequences and 64 tRNA and 17 rRNA were identified. The phylogenetic analysis confirmed that it belongs to the genus *Lentibacillus*. In addition, there were genes related to antibiotic resistance and virulence, and genes predicted as CRISPR and prophage were also identified. Genes related to osmotic stress were found according to the characteristics of halophilic bacterium. Genomic differences from other *Lentibacillus* species were also confirmed through comparative genomic analysis.

**Conclusions:**

Strain CBA3610 is predicted to be a novel candidate species of *Lentibacillus* through phylogenetic analysis and comparative genomic analysis with other species in the same genus. This strain has antibiotic resistance gene and pathogenic genes. In future, the information derived from the results of several genomic analyses of this strain is thought to be helpful in identifying the relationship between halophilic bacteria and human gut microbiota.

**Supplementary Information:**

The online version contains supplementary material available at 10.1186/s13099-021-00436-2.

## Background

*Lentibacillus* is a gram-variable, aerobic or facultatively anaerobic, and halophilic bacterial genus of the family Bacillaceae in the phylum Firmicutes [1]. This genus has been classified as a new genus and species, different from the genus *Virgibacillus*, *Salibacillus*, *Gracilibacillus,* and *Halobacillus*, which was identified to have close phylogenetic relationship, based on 16S rRNA gene sequence analysis and phenotypic characteristics, such as unique lipid content and fatty acid profile [2]. The presence of halophilic prokaryotes in the human gut has been confirmed by various molecular biological and next-generation sequencing (NGS) techniques. However, little had been known about the information of halophilic microorganisms inhabiting the human gut [3]. Recently, halophilic microorganisms have been isolated and reported through development of culturomics [4, 5]. The previous study suggested that the presence of halophilic microbiota in the gut is associated with high salinity in the gut. High salinity of human gut changes the halophilic microbiota which could be related to human diseases such as obesity [5]. Therefore, further studies of halophilic bacteria isolated from the human gut could be helpful in elucidating the relationship between halophilic bacteria and human health. We isolated a bacterium belonging to the *Lentibacillus* species from human fecal sample, identified its whole genome sequence through NGS, and analyzed information on the genes that could have a pathogenic effect on humans. In addition, we performed phylogenetic analysis based on 16S rRNA gene sequence and comparative genomic analysis with other species of genus *Lentibacillus*.

## Methods

### Bacterial strain isolation

Strain CBA3610 was isolated from a stool sample from a 28-year-old healthy male in Gwangju, Republic of Korea. The fecal sample was enriched in Deutsche Sammlung von Mikroorganismen und Zellkulturen (DSMZ) medium 372 broth under aerobic conditions at 37 °C for 7 days, after which 100 mL of the enriched broth was spread on DSMZ medium 372 agar plates to isolate bacterial strains under aerobic conditions at 37 °C for 24 h. Strain CBA3610 was isolated from several colonies, and subculturing was performed under the same conditions at least three times.

### Genome sequencing, assembly, and gene annotation

The genomic DNA of the isolated strain was extracted and purified using the MG genomic DNA purification kit (MGmed, Seoul, Korea). The whole genome sequencing was performed using Pacific Biosciences RS II (Pacific Biosciences, Menlo Park, CA) and Illumina HiSeq X Ten (Illumina, San Diego, CA). Each library used for sequencing was constructed using a 20-kb SMRTbell template preparation kit and a TruSeq Nano DNA High Throughput Library kit. The genome was assembled using the protocol of Unicycler ver. 0.4.6 with PacBio SMRT analysis ver. 2.3 [6] and Pilon ver. 1.21 with Illumina HiSeq for error correction [7]. The subread filtering of the PacBio sequences was performed based on the following criteria: minimum subread length 50, minimum polymerase read quality 75, and minimum polymerase read length 50. Adapter/primer contamination of HiSeq raw sequences was confirmed using FastQC (v0.11.9). The genome was annotated using the Pathosystems Resource Integration Center (PATRIC; https://www.patricbrc.org/) ver. 3.6.7, the bacterial bioinformatics database and analysis resource [8]. We constructed a phylogenetic tree based on 16S rRNA gene sequences. To construct the phylogenetic tree, the sequences of 16S rRNA gene of strain CBA3610 and related species were aligned using Clustal W [9]. Phylogenetic trees were constructed using MEGA 7, based on the neighbor-joining (NJ) [10], maximum parsimony (MP) [11], and maximum likelihood (ML) [12] algorithms using 1000 bootstrap value [13]. Functional genes were predicted and annotated using Rapid Annotation using Subsystem Technology (RAST; https://rast.nmpdr.org/) [14]. PathogenFinder (https://cge.cbs.dtu.dk/services/PathogenFinder/) was used for predicting pathogenicity towards humans [15]. The presence of Clustered Regularly Interspaced Short Palindromic Repeats (CRISPRs) was detected using the CRISPRfinder server (https://crispr.i2bc.paris-saclay.fr/Server/) [16]. Prophages were confirmed using the PHASTER database (https://phaster.ca/), a phage search tool [17].

### Comparative genomics analysis

Comparative genome analysis was performed using 12 reference strains belonging to the genus *Lentibacillus* along with strain CBA3610. The genome and amino acid sequences of 12 reference strains are available in the GenBank of National Center for Biotechnology Information (NCBI, Accessed 22 September 2020). The list of strains used in the analysis is summarized in Additional file 1: Table S1. Pan-genome analysis was performed using Bacterial Pan Genome Analysis tool (BPGA). The 50% sequence identity cut-off was applied to obtain the core genomes of a total of 13 strains using USEARCH (ver. 9.0) [18]. The core genome tree was constructed with the aligned amino acid sequences of common genes of 13 strains using MAFFT (ver. 7.471) [19] and the MEGA 7 with NJ algorithm [10, 13]. The OrthoANI value was calculated using the Orthologous Average Nucleotide Identity Tool (OAT) provided by EzBioCloud database [20].

### Quality assurance

Before genomic DNA extraction, the single colony of strain CBA3610 was transferred three times in DSMZ medium 372 to obtain pure single colony. After obtaining the whole genome sequence of strain CBA3610, the sequence of the 16S rRNA gene, extracted using RNAmmer 1.21 server, was confirmed through the EzBioCloud database.

## Results and discussion

### Genome characteristics and annotation data

**Fig. 1:**
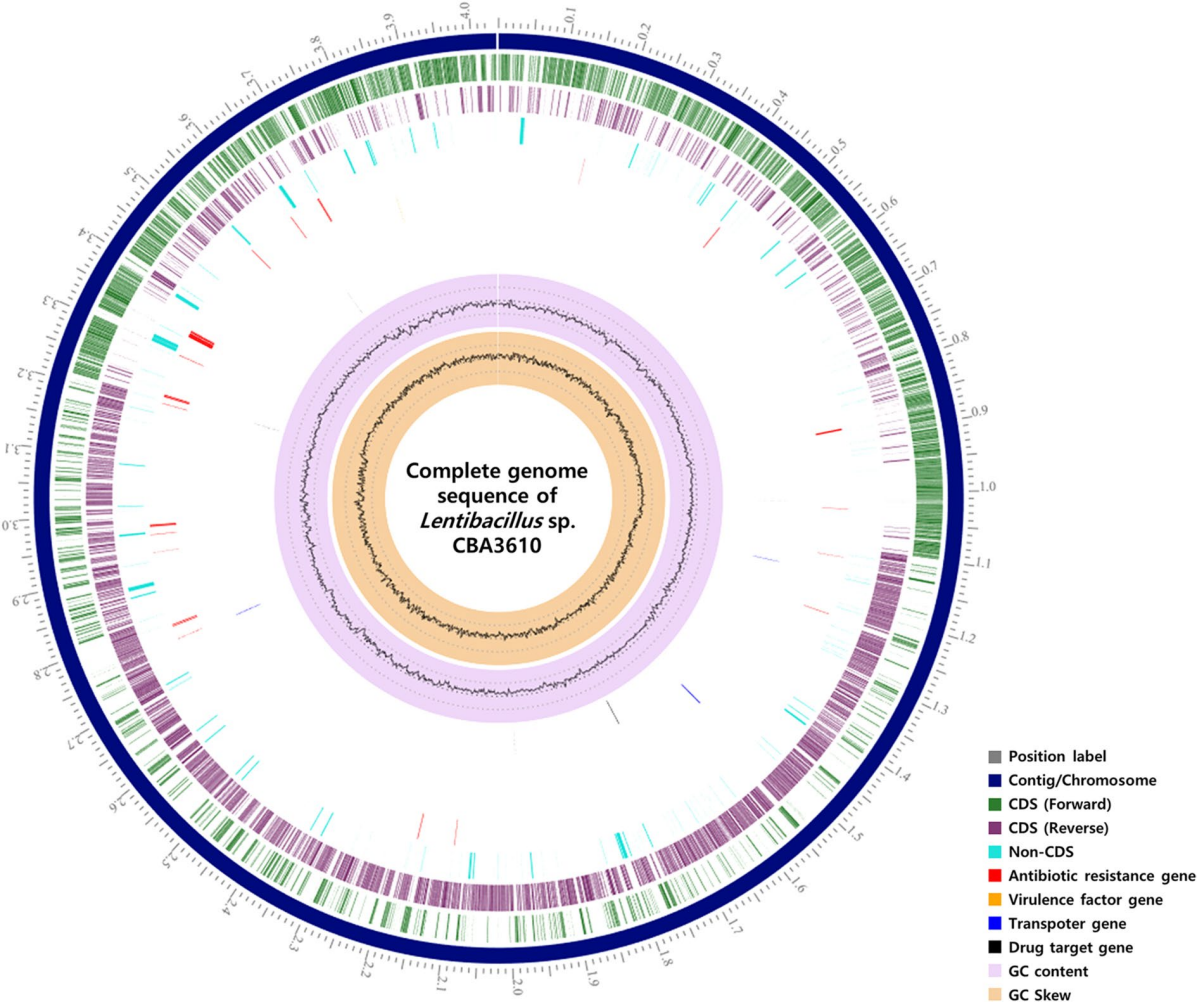
Circular map of *Lentibacillus* sp. CBA3610 genome. From outer to inner rings, the individual circles indicate forward CDS, reverse CDS, non-CDS, antibiotic resistance genes, virulence factor gene, transporter gene, drug target gene, GC content, and GC skew

**Fig. 2:**
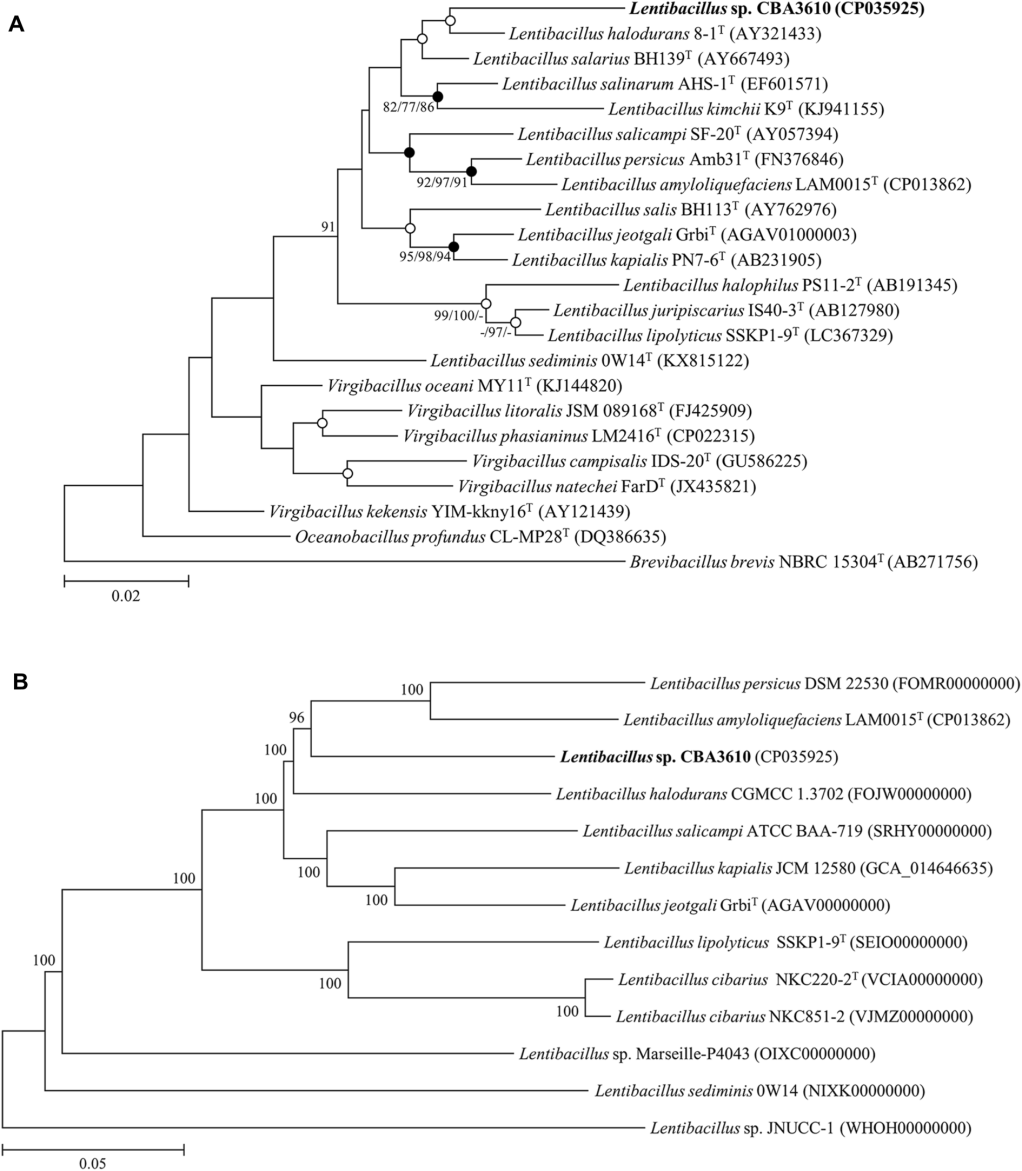
Phylogenetic analysis of strain CBA3610 and reference strains. **A** Phylogenetic tree based on 16S rRNA gene sequences of strain CBA3610 and reference strains. Bootstrap values of the respective neighbor-joining, maximum parsimony, and maximum likelihood (> 70%) are shown at the nodes. Closed and open circles indicate that corresponding branch points were established by both maximum parsimony and maximum likelihood methods, and maximum parsimony or maximum likelihood method, respectively. **B** Phylogenetic tree based on core-genome sequences of strain CBA3610 and reference strains

After the PacBio subreads filtering process, the total number of bases was 1,186,149,844 and the number of reads was 111,990. After the HiSeq raw data filtering process, the total number of bases in the filtered dataset was 796,687,476 and number of reads was 5,276,076. In the de novo assembly process, long-reads of PacBio were assembled using the default option. After de novo assembly with PacBio subreads and error correction using HiSeq reads, the complete genome of *Lentibacillus* sp. CBA3610 consists of one chromosome (Total length: 4,035,571 bp). No plasmid was identified. Chromosome was circular with 42% G + C content. According to the PATRIC annotation results, the genome has 4714 predicted genes, 166 repeat regions, 64 tRNA genes, and 17 rRNA genes. The genome of *Lentibacillus* sp. CBA3610 was annotated as having one virulence factor, four transporters, four drug targets, and 37 antibiotic resistance genes. The circular map of the genome is shown in Fig. 1, and detailed genomic characteristics are listed in Table 1. A phylogenetic tree was constructed, based on the 16S rRNA gene sequences of the strains with close similarity to the *Lentibacillus* sp. CBA3610 (Fig. 2A). The similarity of the 16S rRNA gene sequence of strain CBA3610 with *Lentibacillus salicampi* SF-20^T^, *Lentibacillus salarius* BH139^T^ and *Lentibacillus halodurans* 8-1^T^ was 95.81%, 95.63% and 95.61%, respectively. On the phylogenetic tree based on the 16S rRNA gene sequences, strain CBA3610 clustered with *Lentibacillus halodurans* 8-1^T^ and *Lentibacillus salarius* BH139^T^. Strain CBA3610 can be considered as a novel candidate species of the genus *Lentibacillus* [21]. Based on the results of the RAST annotation, the following categories were classified in the SEED subsystem: amino acids and derivatives (346), carbohydrates (265), protein metabolism (194), cofactors, vitamins, prosthetic groups, and pigments (106) (Additional file 1: Figure S1). Among the 27 categories based on RAST annotation, 55 coding sequences (CDSs) existed in the ‘Virulence, Disease and Defense’. Among these, five CDSs were found to belong to the ‘Resistance to fluoroquinolones’ category related to antibiotic resistance. Based on the results of the PathogenFinder, this strain was not classified as a human pathogen because only one sequence classified as pathogenic, and 14 other sequences classified as non-pathogenic, were identified (Additional file 1: Table S2). The sequence classified as that belonging to the pathogenic family showed 84.78% similarity to those annotated with the function of 30S ribosomal protein S19 in the genome of *Listeria monocytogenes* 08-5578. CRISPRFinder detected five sequences presumed to be CRISPR candidates (Additional file 1: Table S3), and two incomplete prophage regions were found using PHASTER (Additional file 1: Table S4). Among the incomplete prophage regions, region 1 was confirmed to match PHAGE_Bacill_G_NC_023719 and region 2 matched to PHAGE_Brevib_Jimmer1_NC_029104.

**Table 1:** Complete genome features of *Lentibacillus* sp. CBA3610

Property	Value
Sequencing platform	PacBio RS II
Topology	Circular
Genome size (bp)	4,035,571
Contig numbers	1
Genome coverage (fold)	242
DNA G + C (%)	42.04
Number of tRNA genes	64
Number of rRNA genes	17
Number of CDSs	4714
CRISPR candidates	5

### Osmotic stress-related genes

Taking into consideration of the characteristics of *Lentibacillus* of being a halophilic bacterium that survives in a high salinity environment, genes related to osmotic stress of strain CBA3610 were analyzed. In the SEED subsystem of the RAST server, total 34 genes classified as related to ‘osmotic stress’, were identified. Among these, one gene was classified as related to osmoregulation and the remaining 33 genes were annotated to be related to choline and betaine uptake and betaine biosynthesis. The gene involved in osmoregulation encoded the aquaporin family protein, a transporter of glycerol across the cytoplasmic membrane that has limited permeability to small uncharged compounds, such as water. The remaining 33 genes are described as follows. As genes involved in the biosynthesis of osmoprotectant glycine betaine, there is one *betA* gene that encodes oxygen-dependent choline dehydrogenase, which converts choline to betaine aldehyde, and three *betB* genes that encode NAD/NADP-dependent betaine aldehyde dehydrogenase, which converts betaine aldehyde to glycine betaine. In addition, there are seven *opuD* genes that encode glycine betaine transporter, which are involved in glycine betaine uptake, and 12 genes belonging to the *opuA* gene family (including the *opuAA*, *opuAB*, and *opuAC* gene) that encode glycine betaine/carnitine/choline ABC transporter. Lastly, there are two *ProV* genes encoding glycine betaine/proline betaine transport system ATP-binding protein involved in glycine betaine and proline betaine uptake, four genes belonging to the *opuB* gene cluster, including *opuBA*, *opuBB*, *opuBC*, and *opuBD* genes, encoding glycine betaine/carnitine/choline ABC transporter, one *opuCB* gene encoding carnitine transport permease protein, and three *soxA* genes that encode sarcosine oxidase alpha subunit that converts sarcosine to glycine [22–24].

### Comparative genomics

Results of the pan-genome analysis using BPGA showed that between strain CBA3610 and 12 reference strains, 11,961 genes of pan-genome and 849 genes of core genome were found (Additional file 1: Figure S2). Strain CBA3610 had 2212 accessory genes (present in genome of 2–12 strains) of strain CBA3610 and 449 unique genes present only in genome (Additional file 1: Table S5). In the phylogenetic tree based on the core genome, strain CBA3610 was located close to *Lentibacillus persicus*, *Lentibacillus amyloliquefaciens*, and *Lentibacillus halodurans*; it was confirmed that strain CBA3610 belongs to the genus *Lentibacillus* (Fig. 2B). The calculated OrthoANI values between strain CBA3610 and the remaining 12 reference strains are summarized in Additional file 1: Table S6. The range of OrthoANI values between strain CBA3610 and the remaining 12 reference strains was 68.69–79.68%, showing the minimum value with strain *Lentibacillus* sp. JNUCC-1 and the maximum value with strain *Lentibacillus halodurans* CGMCC 1.3702. This result also supplements that of the phylogenetic analysis described above.

## Conclusion

The sequencing process to obtain the genome of *Lentibacillus* sp. CBA3610 and general characteristics of the genome were summarized, and additional genomic characteristics were analyzed using various databases. It is predicted that the probability of strain CBA3610 having a pathogenic effect on humans is low. However, considering the ongoing studies to elucidate the relationship between the human gut microbiome and halophilic microbiota, we believe that this genome information may be helpful in future studies.

## Supplementary Information

**Additional file 1: Figure S1.** Subsystem distribution of *Lentibacillus* sp. CBA3610 genome using SEED analysis. **Figure S2.** Pan- and core-genome box plots of *Lentibacillus *sp. CBA3610 and 12 reference *Lentibacillus* strains with standard deviations. **Table S1.** List of strains used in pan-genomic analysis. **Table S2.** PathogenFinder results of *Lentibacillus* sp. CBA3610. **Table S3.** CRISPR candidate sequences of *Lentibacillus* sp. CBA3610. **Table S4.** Prophages of *Lentibacillus* sp. CBA3610. **Table S5.** The numbers of core-, accessory-, and unique genes of *Lentibacillus *sp. CBA3610 and 12 reference strains. **Table S6.** OrthoANI values between strain CBA3610 and 12 reference *Lentibacillus* strains.

## Data Availability

The complete genome data of *Lentibacillus* sp. CBA3610 has been deposited in DDBJ/EMBL/GenBank, with Accession Number CP035925.
