# Supplementary material for: Genomic analysis of halophilic bacterium, Lentibacillus sp. CBA3610, derived from human feces
Source: Gut Pathog. 2021 Jun 23;13:41. doi: 10.1186/s13099-021-00436-2 (PMC8220782; doi:10.1186/s13099-021-00436-2)
Supplement: Supplementary file 1 — Additional file 1: Figure S1. Subsystem distribution of Lentibacillus sp. CBA3610 genome using SEED analysis. Figure S2. Pan- and core-genome box plots of Lentibacillus sp. CBA3610 and 12 reference Lentibacillus strains with standard deviations. Table S1. List of strains used in pan-genomic analysis. Table S2. PathogenFinder results of Lentibacillus sp. CBA3610. Table S3. CRISPR candidate sequences of Lentibacillus sp. CBA3610. Table S4. Prophages of Lentibacillus sp. CBA3610. Table S5. The numbers of core-, accessory-, and unique genes of Lentibacillus sp. CBA3610 and 12 reference strains. Table S6. OrthoANI values between strain CBA3610 and 12 reference Lentibacillus strains. [file 13099_2021_436_MOESM1_ESM.docx]

**Additional file 1: Figure S1.** Subsystem distribution of *Lentibacillus* sp. CBA3610 genome using SEED analysis.

**
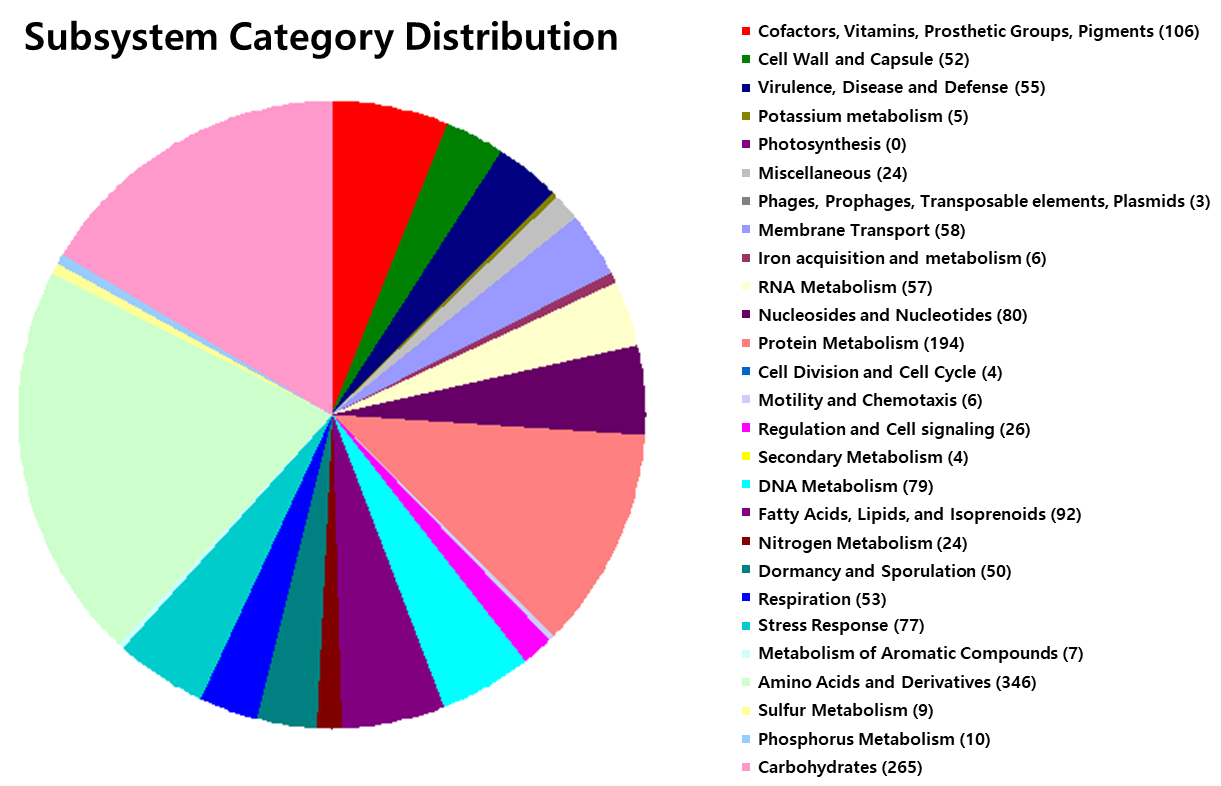
**

**Additional file 1: Figure S2.** Pan- and core-genome box plots of *Lentibacillus* sp. CBA3610 and 12 reference *Lentibacillus* strains with standard deviations.

**
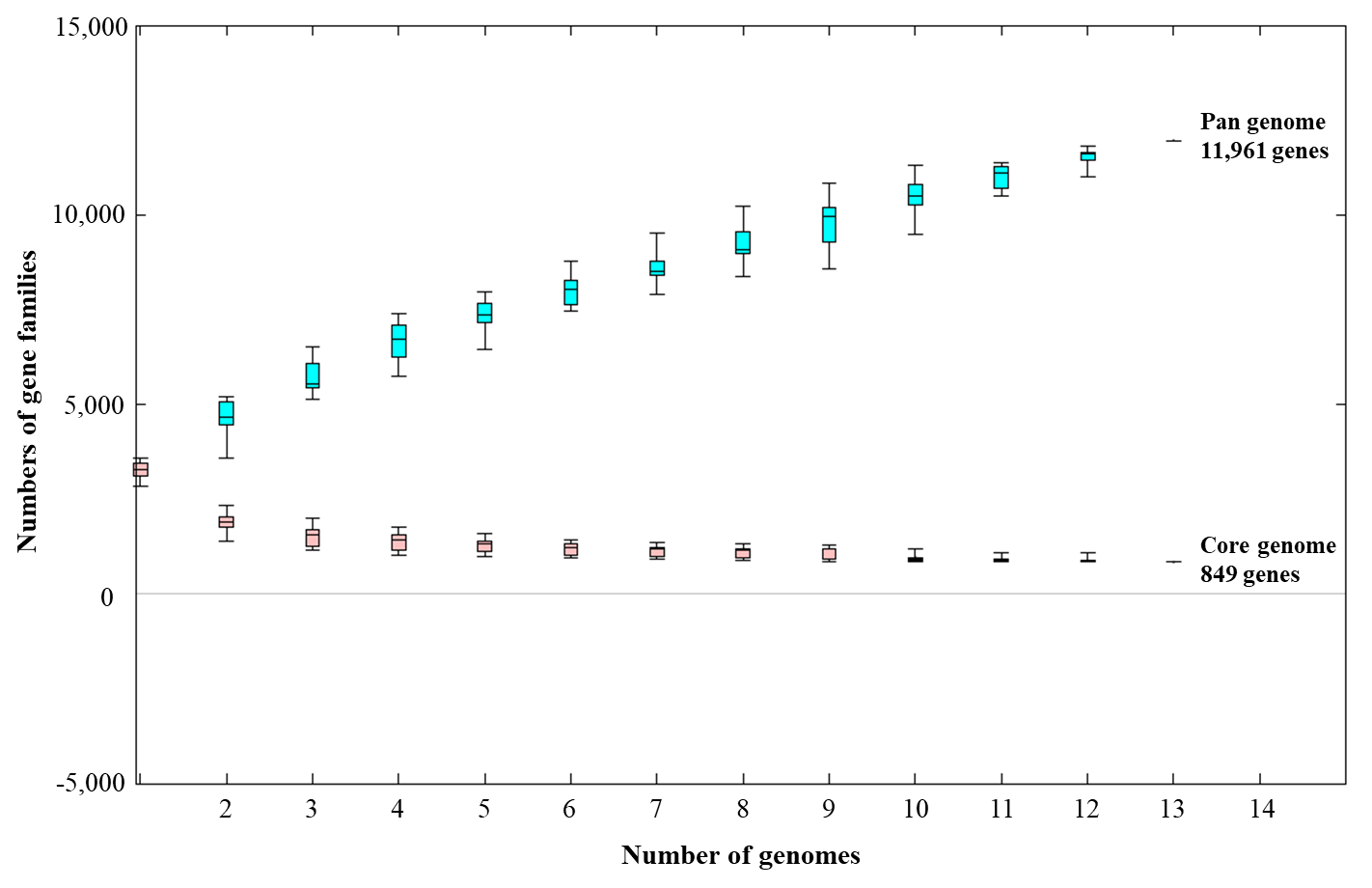
**

**Additional file 1: Table S1.** List of strains used in pan-genomic analysis

| **No.** | **Strain name** | **NCBI accession no.** | **Genome status (Number of contigs)** |
| --- | --- | --- | --- |
| 1 | *Lentibacillus* sp. CBA3610 | CP035925 | Complete (1) |
| 2 | *Lentibacillus kapialis* JCM 12580 | GCA_014646635 | Scaffold (148) |
| 3 | *Lentibacillus persicus* DSM 22530 | FOMR00000000 | Scaffold (23) |
| 4 | *Lentibacillus* *amyloliquefaciens* LAM0015^T^ | CP013862 | Complete (1) |
| 5 | *Lentibacillus* *halodurans* CGMCC 1.3702 | FOJW00000000 | Scaffold (31) |
| 6 | *Lentibacillus jeotgali* Grbi^T^ | AGAV00000000 | Contig (31) |
| 7 | *Lentibacillus salicampi* ATCC BAA-719 | SRHY00000000 | Contig (135) |
| 8 | *Lentibacillus sediminis* 0W14 | NIXK00000000 | Contig (34) |
| 9 | *Lentibacillus* sp. JNUCC_1 | WHOH00000000 | Contig (3) |
| 10 | *Lentibacillus* sp. Marseille P4043 | OIXC00000000 | Scaffold (3) |
| 11 | *Lentibacillus* *cibarius* NKC220-2^T^ | VCIA00000000 | Contig (3) |
| 12 | *Lentibacillus* *cibarius* NKC851-2 | VJMZ00000000 | Contig (5) |
| 13 | *Lentibacillus* *lipolyticus* SSKP1-9^T^ | SEIO00000000 | Scaffold (153) |

**Additional file 1: Table S2.** PathogenFinder results of *Lentibacillus* sp. CBA3610

| No. | Input Sequence | Accession ID^a^ | Organisms | Class | Protein fuction | Protein ID^b^ | %Identity | Matched Family |
| --- | --- | --- | --- | --- | --- | --- | --- | --- |
| 1 | contig1_3412 # 3353148 # 3353426 # 1 # ID=1_3412;partial=00;start_type=ATG;rbs_motif=AGGAGG;rbs_spacer=5-10bp;gc_cont=0.416 | CP001602 | Listeria monocytogenes 08-5578, complete genome. | Bacillales | 30S ribosomal protein S19 | ADB66989 | 84.78 | Matched Pathogenic Families |
| 2 | contig1_1358 # 1290087 # 1290554 # -1 # ID=1_1358;partial=00;start_type=TTG;rbs_motif=AGGA;rbs_spacer=5-10bp;gc_cont=0.408 | BA000004 | Bacillus halodurans C-125 DNA, complete genome. | Bacillales | ribonucleoside-diphosphate reductase (major subunit) | BAB06529 | 81.29 | Matched Not Pathogenic Families |
| 3 | contig1_3293 # 3224183 # 3225649 # 1 # ID=1_3293;partial=00;start_type=ATG;rbs_motif=AGGAGG;rbs_spacer=5-10bp;gc_cont=0.453 | BA000028 | Oceanobacillus iheyensis HTE831 DNA, complete genome. | Bacillales | inosine-5'-monophosphate dehydrogenase | BAC11966 | 80.74 | Matched Not Pathogenic Families |
| 4 | contig1_2086 # 1996553 # 1997554 # -1 # ID=1_2086;partial=00;start_type=TTG;rbs_motif=GGAG/GAGG;rbs_spacer=5-10bp;gc_cont=0.433 | BA000028 | Oceanobacillus iheyensis HTE831 DNA, complete genome. | Bacillales | dipeptide ABC transporter permease | BAC14406 | 83.18 | Matched Not Pathogenic Families |
| 5 | contig1_645 # 615998 # 616840 # 1 # ID=1_645;partial=00;start_type=ATG;rbs_motif=AGxAGG/AGGxGG;rbs_spacer=5-10bp;gc_cont=0.490 | CP001794 | Geobacillus sp. Y412MC61, complete genome. | Bacillales | short-chain dehydrogenase/reductase SDR | ACX77409 | 81.43 | Matched Not Pathogenic Families |
| 6 | contig1_2700 # 2655343 # 2655483 # -1 # ID=1_2700;partial=00;start_type=GTG;rbs_motif=GGAG/GAGG;rbs_spacer=5-10bp;gc_cont=0.504 | AP006627 | Bacillus clausii KSM-K16 DNA, complete genome. | Bacillales | 2-hydroxyhepta-2,4-diene-1,7-dioate isomerase | BAD64708 | 82.61 | Matched Not Pathogenic Families |
| 7 | contig1_3037 # 2994916 # 2995596 # -1 # ID=1_3037;partial=00;start_type=ATG;rbs_motif=3Base/5BMM;rbs_spacer=13-15bp;gc_cont=0.391 | BA000028 | Oceanobacillus iheyensis HTE831 DNA, complete genome. | Bacillales | hypothetical protein | BAC15066 | 80.97 | Matched Not Pathogenic Families |
| 8 | contig1_419 # 406985 # 407497 # 1 # ID=1_419;partial=00;start_type=ATG;rbs_motif=AGGAGG;rbs_spacer=5-10bp;gc_cont=0.388 | BA000028 | Oceanobacillus iheyensis HTE831 DNA, complete genome. | Bacillales | hypothetical conserved protein | BAC12659 | 87.06 | Matched Not Pathogenic Families |
| 9 | contig1_3415 # 3354498 # 3354884 # 1 # ID=1_3415;partial=00;start_type=ATG;rbs_motif=None;rbs_spacer=None;gc_cont=0.452 | CP000485 | Bacillus thuringiensis str. Al Hakam, complete genome. | Bacillales | LSU ribosomal protein L16P | ABK83530 | 86.72 | Matched Not Pathogenic Families |
| 10 | contig1_729 # 695538 # 695987 # -1 # ID=1_729;partial=00;start_type=ATG;rbs_motif=GGAGG;rbs_spacer=5-10bp;gc_cont=0.422 | BA000028 | Oceanobacillus iheyensis HTE831 DNA, complete genome. | Bacillales | transcriptional regulator (Lrp/AsnC family) | BAC15227 | 83.22 | Matched Not Pathogenic Families |
| 11 | contig1_1027 # 975340 # 975702 # 1 # ID=1_1027;partial=00;start_type=ATG;rbs_motif=AGGAG;rbs_spacer=5-10bp;gc_cont=0.446 | CP000817 | Lysinibacillus sphaericus C3-41, complete genome. | Bacillales | Chemotaxis protein cheY-like protein | ACA39162 | 80.83 | Matched Not Pathogenic Families |
| 12 | contig1_1361 # 1293614 # 1293985 # 1 # ID=1_1361;partial=00;start_type=ATG;rbs_motif=GGxGG;rbs_spacer=5-10bp;gc_cont=0.411 | BA000028 | Oceanobacillus iheyensis HTE831 DNA, complete genome. | Bacillales | hypothetical conserved protein | BAC13857 | 81.3 | Matched Not Pathogenic Families |
| 13 | contig1_1950 # 1855530 # 1855754 # 1 # ID=1_1950;partial=00;start_type=ATG;rbs_motif=AGGAG;rbs_spacer=5-10bp;gc_cont=0.458 | AL009126 | Bacillus subtilis subsp. subtilis str. 168 complete genome. | Bacillales | putative iron-sulfur scaffold protein | CAB15212 | 81.08 | Matched Not Pathogenic Families |
| 14 | contig1_1164 # 1110708 # 1110947 # -1 # ID=1_1164;partial=00;start_type=TTG;rbs_motif=GGAGG;rbs_spacer=5-10bp;gc_cont=0.392 | BA000028 | Oceanobacillus iheyensis HTE831 DNA, complete genome. | Bacillales | ferredoxin [3Fe-4S][4Fe-4S] | BAC13686 | 82.28 | Matched Not Pathogenic Families |
| 15 | contig1_2218 # 2134716 # 2134955 # -1 # ID=1_2218;partial=00;start_type=GTG;rbs_motif=AGGAGG;rbs_spacer=5-10bp;gc_cont=0.471 | BA000028 | Oceanobacillus iheyensis HTE831 DNA, complete genome. | Bacillales | ferredoxin | BAC14536 | 82.28 | Matched Not Pathogenic Families |

^a^NCBI accession ID

^b^NCBI protein ID

**Additional file 1: Table S3.** CRISPR candidate sequences of *Lentibacillus* sp. CBA3610

| CRISPR candidate | Locus | CRISPR length | DR consensus | DR length | Number of spacer |
| --- | --- | --- | --- | --- | --- |
| Possible CRISPR 1 | (728317...728393) | 76 | GCTTCTCTCGCTGGGAATCCAGCA | 24 | 1 |
| Possible CRISPR 2 | (1825318...1825495) | 177 | GAGAAGGCCTCTCGGTGACCTTT | 23 | 3 |
| Possible CRISPR 3 | (2248539...2248624) | 85 | TCAGGAGGTCATAGCACTTTCTGATC | 26 | 1 |
| Possible CRISPR 4 | (3642411...3642489) | 78 | CTTTTCTGCGAGGAGTGCTGCATC | 24 | 1 |
| Possible CRISPR 5 | (3664198...3664287) | 89 | ATTGCATGTATGCAAGTAATTTTTT | 25 | 1 |

DR: highly conserved regions

**Additional file 1: Table S4.** Prophages of *Lentibacillus* sp. CBA3610

| Region | Region Length | Completeness* | Score | # Total Proteins | Region Position | Most Common Phage | GC % |
| --- | --- | --- | --- | --- | --- | --- | --- |
| 1 | 29.3Kb | incomplete | 40 | 11 | 3641511-3670900 | PHAGE_Bacill_G_NC_023719(2) | 42.67% |
| 2 | 28.6Kb | incomplete | 40 | 27 | 3733541-3762206 | PHAGE_Brevib_Jimmer1_NC_029104(1) | 37.74% |

*Completeness: Incomplete (score < 70)

Region: The number assigned to the region.

Region Length: The length of the sequence of that region (in bp).

Completeness: A prediction of whether the region contains a intact or incomplete prophage based on the above criteria.

Score: The score of the region based on the above criteria.

# Total Proteins: The number of ORFs present in the region.

Region Position: The start and end positions of the region on the bacterial chromosome.

Most Common Phage: The phage(s) with the highest number of proteins most similar to those in the region.

GC %: The percentage of GC nucleotides of the region.

**Additional file 1: Table S5.** The numbers of core-, accessory-, and unique genes of *Lentibacillus* sp. CBA3610 and 12 reference strains

| No. | Organism name | No. of  core genes | No. of  accessory genes | No. of  unique genes |
| --- | --- | --- | --- | --- |
| 1 | *Lentibacillus* sp. CBA3610 | 849 | 2121 | 449 |
| 2 | *Lentibacillus kapialis* JCM 12580 | 849 | 2029 | 526 |
| 3 | *Lentibacillus persicus* DSM 22530 | 849 | 2024 | 266 |
| 4 | *Lentibacillus* *amyloliquefaciens* LAM0015^T^ | 849 | 2229 | 364 |
| 5 | *Lentibacillus* *halodurans* CGMCC 1.3702 | 849 | 2075 | 358 |
| 6 | *Lentibacillus jeotgali* Grbi^T^ | 849 | 2104 | 310 |
| 7 | *Lentibacillus salicampi* ATCC BAA-719 | 849 | 2243 | 481 |
| 8 | *Lentibacillus sediminis* 0W14 | 849 | 1694 | 917 |
| 9 | *Lentibacillus* sp. JNUCC_1 | 849 | 1194 | 958 |
| 10 | *Lentibacillus* sp. Marseille P4043 | 849 | 1812 | 924 |
| 11 | *Lentibacillus* *cibarius* NKC220-2^T^ | 849 | 1965 | 301 |
| 12 | *Lentibacillus* *cibarius* NKC851-2 | 849 | 1961 | 153 |
| 13 | *Lentibacillus* *lipolyticus* SSKP1-9^T^ | 849 | 1789 | 209 |

**Additional file 1: Table S6.** OrthoANI values between strain CBA3610 and 12 reference *Lentibacillus* strains

|  | 1 | 2 | 3 | 4 | 5 | 6 | 7 | 8 | 9 | 10 | 11 | 12 | 13 |
| --- | --- | --- | --- | --- | --- | --- | --- | --- | --- | --- | --- | --- | --- |
| 1 |  | 77.01 | 76.86 | 79.28 | 79.68 | 79.17 | 78.98 | 70.69 | 68.69 | 71.18 | 73.95 | 74.32 | 74.04 |
| 2 | 77.01 |  | 75.08 | 77.19 | 76.78 | 81.98 | 78.38 | 69.89 | 68.30 | 70.42 | 73.13 | 73.27 | 73.33 |
| 3 | 76.86 | 75.08 |  | 81.66 | 75.62 | 76.06 | 75.46 | 69.61 | 68.19 | 70.12 | 72.54 | 72.88 | 72.65 |
| 4 | 79.28 | 77.19 | 81.66 |  | 77.56 | 79.08 | 77.55 | 70.30 | 68.92 | 70.82 | 73.73 | 73.99 | 73.56 |
| 5 | 79.68 | 76.78 | 75.62 | 77.56 |  | 77.99 | 78.17 | 70.34 | 68.65 | 71.17 | 74.11 | 74.23 | 74.51 |
| 6 | 79.17 | 81.98 | 76.06 | 79.08 | 77.99 |  | 80.65 | 70.49 | 68.67 | 70.83 | 74.07 | 74.00 | 73.81 |
| 7 | 78.98 | 78.38 | 75.46 | 77.55 | 78.17 | 80.65 |  | 70.33 | 68.67 | 70.69 | 73.85 | 73.93 | 73.91 |
| 8 | 70.69 | 69.89 | 69.61 | 70.30 | 70.34 | 70.49 | 70.33 |  | 68.26 | 69.79 | 69.62 | 69.63 | 69.83 |
| 9 | 68.69 | 68.30 | 68.19 | 68.92 | 68.65 | 68.67 | 68.67 | 68.26 |  | 68.80 | 68.90 | 68.85 | 68.60 |
| 10 | 71.18 | 70.42 | 70.12 | 70.82 | 71.17 | 70.83 | 70.69 | 69.79 | 68.80 |  | 70.59 | 70.76 | 70.29 |
| 11 | 73.95 | 73.13 | 72.54 | 73.73 | 74.11 | 74.07 | 73.85 | 69.62 | 68.90 | 70.59 |  | 97.37 | 79.69 |
| 12 | 74.32 | 73.27 | 72.88 | 73.99 | 74.23 | 74.00 | 73.93 | 69.63 | 68.85 | 70.76 | 97.37 |  | 79.76 |
| 13 | 74.04 | 73.33 | 72.65 | 73.56 | 74.51 | 73.81 | 73.91 | 69.83 | 68.60 | 70.29 | 79.69 | 79.76 |  |

1: *Lentibacillus* sp. CBA3610

2: *Lentibacillus kapialis* JCM 12580

3: *Lentibacillus persicus* DSM 22530

4: *Lentibacillus amyloliquefaciens* LAM0015^T^

5: *Lentibacillus halodurans* CGMCC 1.3702

6: *Lentibacillus* *jeotgali* Grbi^T^

7: *Lentibacillus* *salicampi* ATCC BAA-719

8: *Lentibacillus* *sediminis* 0W14

9: *Lentibacillus* sp. JNUCC-1

10: *Lentibacillus* sp. Marseille-P4043

11: *Lentibacillus* *cibarius* NKC220-2^T^

12: *Lentibacillus* *cibarius* NKC851-2

13: *Lentibacillus* *lipolyticus* SSKP1-9^T^
